# Patient Privacy Perspectives on Health Information Exchange in a Mental Health Context: Qualitative Study

**DOI:** 10.2196/13306

**Published:** 2019-11-13

**Authors:** Nelson Shen, Lydia Sequeira, Michelle Pannor Silver, Abigail Carter-Langford, John Strauss, David Wiljer

**Affiliations:** 1 Centre for Addiction and Mental Health Toronto, ON Canada; 2 Institute of Health Policy, Management and Evaluation Dalla Lana School of Public Health University of Toronto Toronto, ON Canada; 3 Interdisciplinary Centre for Health and Society University of Toronto Scarborough Scarborough, ON Canada; 4 Canada Health Infoway Toronto, ON Canada; 5 Department of Psychiatry Faculty of Medicine University of Toronto Toronto, ON Canada; 6 University Health Network Toronto, ON Canada

**Keywords:** privacy, health information exchange, health information technology, attitude to health, trust

## Abstract

**Background:**

The privacy of patients with mental health conditions is prominent in health information exchange (HIE) discussions, given that their potentially sensitive personal health information (PHI) may be electronically shared for various health care purposes. Currently, the patient privacy perspective in the mental health context is not well understood because of the paucity of in-depth patient privacy research; however, the evidence suggests that patient privacy perspectives are more nuanced than what has been assumed in the academic and health care community.

**Objective:**

This study aimed to generate an understanding on how patients with mental health conditions feel about privacy in the context of HIE in Canada. This study also sought to identify the factors underpinning their privacy perspectives and explored how their perspectives influenced their attitudes toward HIE.

**Methods:**

Semistructured interviews were conducted with patients at a Canadian academic hospital for addictions and mental health. Guided by the Antecedent-Privacy Concern-Outcome macro-model, interview transcripts underwent deductive and inductive thematic analyses.

**Results:**

We interviewed 14 participants. Their privacy concerns varied, depending on the participant’s privacy experiences and health care perceptions. Media reports of privacy breaches and hackers had little impact on participants’ privacy concerns because of a fatalistic belief that privacy breaches are a reality in the digital age. Rather, direct observations and experiences with the mistreatment of PHI in health care settings caused concern. Decisions to trust others with PHI depended on past experiences with the individual (or institution) and health care needs. Participants had little knowledge of patient privacy rights and legislation but were willing to participate in HIE because of perceived individual and societal benefits.

**Conclusions:**

This study introduces evidence that patients with mental health conditions would support HIE. Participants were pragmatic, supporting HIE because they wanted the best care possible. They also understood that their PHI was critical in supporting the single-payer Canadian health care system. Participant health care experiences informed their privacy perspectives, trust, and PHI sharing attitudes—all accentuating the importance of the patient experience in building trust in HIE. Their lack of knowledge about patient rights and PHI uses highlights the degree of trust they have in the health care system to protect their privacy. These findings suggest that the patient privacy discourse should extend beyond the oft-cited barrier of patient privacy concerns to include discussions about building trust, communicating the benefits of HIE, and improving patient experiences. Although our findings are in the Canadian context, this study highlights the importance of engaging patients in privacy policy discussions, regardless of jurisdiction, to ensure their nuanced perspectives are reflected in policy decisions on their PHI.

## Introduction

### Privacy and Health Information Exchange

Privacy and trust are critical for patients with mental health conditions. Effective therapeutic patient-provider relationships require patient candor and trust that health care providers will protect patient privacy (or confidentiality) [1,2]. Mental health records often contain sensitive information, including intimate revelations or references to stigmatic medical conditions [3]. As such, people with mental health conditions may be concerned about the disclosure of this sensitive personal health information (PHI). Fear of the stigma and discrimination may cause them to withhold information from health care providers or avoid seeking care altogether—which can be detrimental to patient care [4]. A recent meta-analysis [5] found fear of stigma had a small- to moderate-sized negative effect on health-seeking behavior, and concerns with PHI disclosure was the most commonly reported reason for health care avoidance. For this reason, mental health care and mental health records have historically been isolated from other medical care to protect patient privacy [6,7].

Patient privacy is an issue that has come to the forefront in discussions about health information exchange (HIE) [8]. In this paper, HIE refers to the process where PHI is electronically shared between health care providers, patients, and other health care stakeholders through interoperable health information technology (HIT) [9,10]. HIE can provide HIT users with the best information possible for 3 common uses: clinical care [11], patient access and management of their PHI (ie, patient-mediated exchange) [11-13], and research and health system planning [14-17]. Internationally, there is consensus that HIE can improve health care quality, safety, and efficiency [18,19].

In recognition of the transformative potential of HIE, Canadian federal and provincial or territorial governments have made significant investment into the creation of interoperable HIT (ie, electronic health records [EHRs]) to enable HIE to support their single-payer, publicly funded *universal* health care system—an institution rooted in the Canadian identity [19,20]. Despite the strong interest, the adoption of HIE in Canada has been slow [21]. Privacy is an oft-identified adoption barrier, as the seamless flow of PHI creates challenges to protecting patient privacy [8,16,22]. Much of the privacy debate centers around whether HIE would raise patient privacy concerns, erode trust in patient-provider relationships, and cause adverse health care behaviors [23-27].

### Privacy Perspectives of Patients with Mental Health Conditions

From a mental health perspective, there are divergent views on the appropriate use of mental health records and its inclusion in HIE [28-30]. These debates have overshadowed the value of HIE. For instance, the inclusion of psychiatric notes in the EHR were found to reduce hospital readmission rates for psychiatric patients [31]. HIT supporting patient-mediated exchange of PHI via a mental health care patient portal could improve patient activation, patient recovery, and appointment attendance [32]. Finally, population-based research using large databases has been an effective tool in battling the stigmatization of mental health disorders. Evidence generated from research has been used to raise awareness of the societal burden of mental health, identify gaps in treatment efficacy and effectiveness, and increase access to mental health care through more efficient utilization of health care resources [33].

The balance between protecting patient privacy and providing optimal care is value-laden, requiring careful consideration of all stakeholder perspectives. Unfortunately, the patient perspective is often based on conjecture, reflecting the values and norms of the academic and health care community [34]. Sometimes patient privacy needs are overestimated [35-37]. A 2018 systematic review found the patient privacy perspective was more nuanced and context dependent than what was suggested [38]. An emerging stream of research suggests that patient-perceived benefits can offset the postulated impact of privacy concerns [39-48]. This privacy trade-off is known as the privacy calculus—a cognitive risk-benefit analysis used to determine their information sharing behavior [49]. There is evidence of this trade-off for patients with sensitive PHI [45-48] but not specifically in the mental health context [38]. With policy makers trying to overcome the challenges of HIT for mental health [50], we need a better understanding of the patient privacy perspective to ensure patient-centered policy decisions are made [51,52]. The aim of this study was to generate insights on how patients with mental health conditions feel about privacy in the context of HIE.

## Methods

### Theoretical Framework

**Figure 1:**
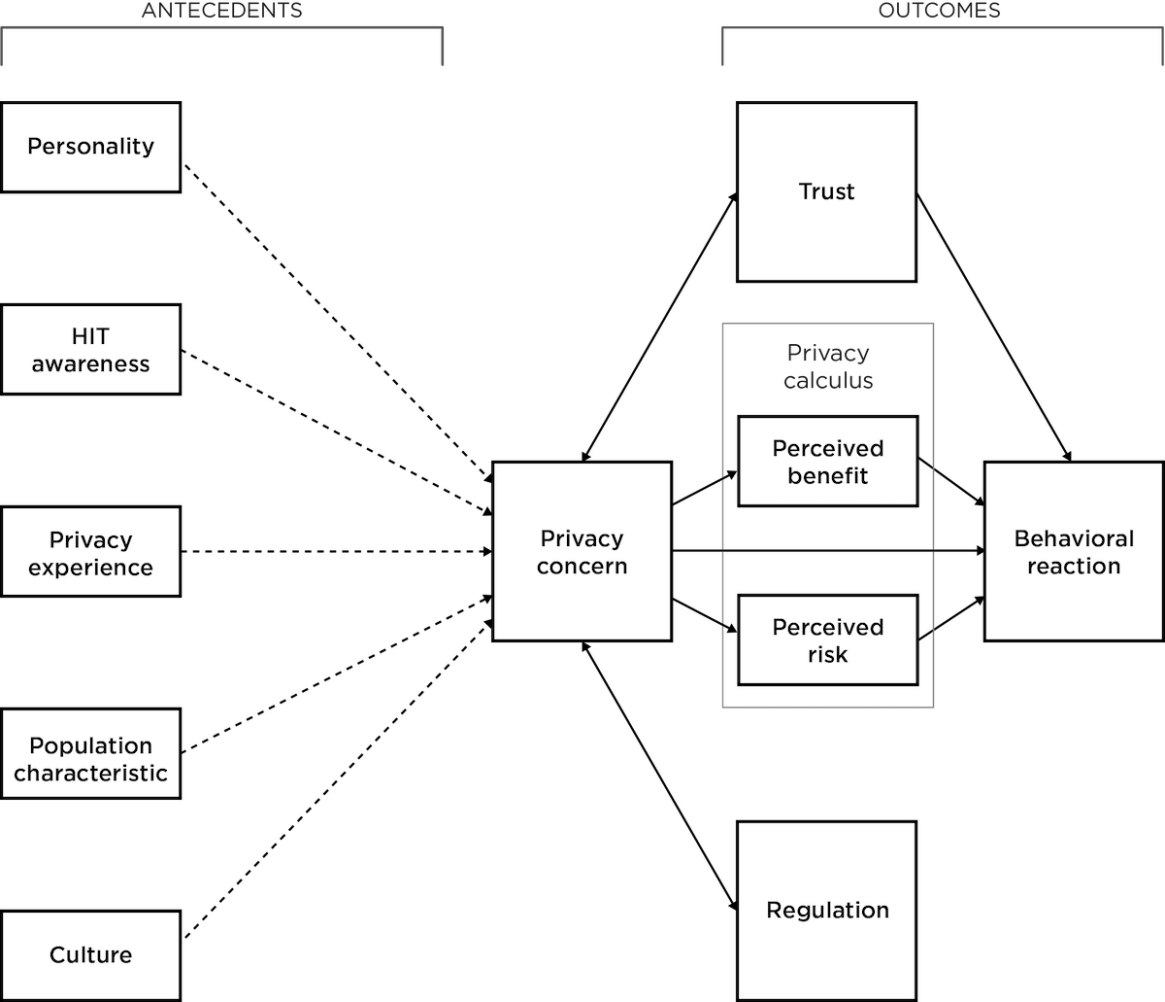
Adapted Antecedent-Privacy Concern-Outcome model. Dotted arrows indicate tenuous relationships between constructs (ie, has not been confirmed through repeated studies).

This study is a part of a larger project aimed at adapting the Antecedent-Privacy Concern-Outcome (APCO) macro-model [53] for use in health informatics research. The APCO is a high-level process model that delineates how antecedents contribute to privacy concern and how concerns can impact information sharing behaviors. *Behavioral reaction* is the most prominent outcome, as it represents an individual’s intention to use a Web-based service or technology. *Regulation* and *trust* are proposed to have reciprocal relationships with *privacy concern*, acting as both antecedents and outcomes. The privacy calculus is included as *perceived risk* and *perceived benefit*. An adapted APCO model (Figure 1) was used as the framework for this study [38]. Its constructs (herein *italicized*) are presented and defined in Table 1.

With the dearth of in-depth and qualitative patient privacy research in mental health [38], this study was conducted to bridge this evidence gap. The objective of this study was to understand how patients with mental health conditions feel about privacy as it relates to their PHI and its uses facilitated through HIE (ie, clinical use, secondary use, and patient-mediated exchange). This study also sought to identify the factors underpinning their privacy perspectives and explore how their perspectives influence their willingness to electronically share PHI through HIE. Using the APCO as a guiding framework, we asked the following questions:

- How do patients feel about the privacy of their PHI (privacy concern)?
- What are the reasons for their privacy perspective (APCO antecedents)?
- Who do patients trust with their PHI (trust)? Why?
- What is the role of privacy policies and regulations (regulations) in the patient privacy perspective?
- What do patients know about their PHI rights and the legislated PHI uses (regulations)? How do they feel about them?
- How do patients feel about the various uses of PHI via HIE (behavioral reaction)?

**Table 1:** Adapted Antecedent-Privacy Concern-Outcome construct definitions.

Antecedent-Privacy Concern-Outcome domain and construct		Definition
Privacy concern		An individual’s beliefs, attitudes, and concerns about the electronic sharing of their PHI^a^
**Antecedent**		
	Privacy experience	The extent to which individuals have been exposed to or been a victim of information abuses
	HIT^b^ awareness	The extent to which individuals have been exposed to or have knowledge of HIT
	Population characteristic	The differences based on the shared characteristics of a population (eg, age, gender, income, education, etc)
	Personality	An individual’s psychological characteristics, patterns of thinking, feeling, and behaving
	Culture	The attitudes, customs, and beliefs that distinguish one group of people from another
**Outcome**		
	Perceived benefit	The degree to which an individual believes that the electronic sharing of their PHI can help themselves and others
	Perceived risk	The degree to which an individual believes that the electronic sharing of their PHI will result in a loss or harm
	Behavioral reaction	An individual’s intention to electronically share their PHI or use HIT
**Antecedent and outcome**		
	Trust	An individual’s willingness to become vulnerable to the actions of another party
	Regulation	An individual’s knowledge of and attitudes toward the privacy safeguards and use of their electronic PHI

^a^PHI: personal health information.

^b^HIT: health information technology.

### Recruitment

This study was conducted at the Centre for Addiction and Mental Health (CAMH)—Canada’s largest academic health sciences center for mental health. Through consultation with the CAMH leadership, we recruited patients receiving acute care and structured treatments from 2 main programs at CAMH: Mood and Anxiety and Addiction Medicine Services. Together, these programs serve CAMH patients with depression; bipolar disorder; anxiety disorders; obsessive-compulsive disorders; and drug, alcohol, gambling, and other addiction issues, accounting for approximately 15,000 patients of the 37,065 unique CAMH patients.

To be eligible for this study, participants had to be receiving care at one of the CAMH programs, English-speaking, ≥18 years, and able to provide written informed consent. Participants were offered a Can $10 coffee gift card and reimbursed for public transportation costs. Research ethics approval (CAMH 067-2015) was acquired before recruitment. Potential participants were invited to participate through clinician referral, advertisements at participating clinics, and the CAMH research study website. Participants were also recruited at the end of patient group meetings, where CAMH researchers were scheduled to provide a 1-min description about their study. The clinicians prefaced and emphasized that the research was independent to treatment program and that participation was voluntary, having no bearing on the care they receive at CAMH. Interested patients could approach the researcher for more information about the study or sign up after the meeting. The lead author (NS) recruited participants from these meetings and introduced the study as a part of his PhD thesis on understanding patient views on privacy.

A maximum variation purposive sampling strategy was employed to identify cross-cutting themes derived from a diverse range of perspectives [54]. This strategy requires the researcher to first identify relevant diversity characteristics as criteria and then choose participants that meet these criteria to provide maximum variation in the data collected [55]. Participants were asked to fill out a preinterview screening questionnaire (Multimedia Appendix 1) and were included or excluded serially, with each included participant contributing a unique background to the study [56]. Variation was sought across the following *population characteristics*: treatment program, years at CAMH, and self-reported health status (Health Utility Index Mark III [57]). A trusting disposition scale [58] was also used to assess an individual’s general propensity to trust others (ie, *personality*). Trusting disposition is based on their willingness to give people a chance until proven wrong (ie, trusting stance) and general belief that people generally act with benevolence, integrity, and competence (ie, faith in humanity). Given the challenges of recruiting participants from this population, especially individuals with distrusting dispositions, participant interview responses regarding to trust (or distrust) was used in conjunction with the trusting disposition scale to ensure the study included a diversity of views on trust.

Recruitment continued until theoretical saturation was achieved (April to June 2017). Saturation was defined as the point where the interviews yielded no new data or themes. An a priori thematic saturation approach was undertaken to exemplify a theory (ie, ACPO) based on its predetermined theoretical constructs [59].

### Data Collection

One-on-one interviews were conducted in-person at CAMH sites, each lasting approximately 45 min. A semistructured interview format was selected for the interviews to allow the interviewer (NS) to diverge and pursue ideas in more depth when necessary [60]. Informed consent was collected before the interviews. NS introduced himself as a PhD candidate, affiliated with CAMH as a research trainee and disclosed that he had no involvement in the delivery of patient care. Participants were reassured that participation was independent from the care they receive at CAMH, and their individual responses would only be accessed by the research team for data analysis. They were also informed that the interviews would be audio recorded for transcription, and field notes would be taken throughout and after the interviews.

An interview guide (Multimedia Appendix 2) was developed by the research team and focused on the patient perspectives on *privacy concern, trust, regulation, and behavioral reaction*. Each section began with a broad question and narrowed down to focus on the *why*, allowing latent concepts to emerge through participant responses [61]. For this reason, specific questions related to privacy antecedents and privacy calculus were not included in the interview guide. The section on regulation also included an educational component where participants were asked broad questions about their views on *regulation* and what they knew about their patient rights and legislated PHI uses. They were then briefed on the provisions pertaining to their rights (ie, access records, request audit, and request consent directives) and permitted PHI uses (ie, use in provision of care, health system planning, and research ethics board [REB]–approved research). With this context, we then asked participants who they trusted with their PHI (*trust*) and whether they were willing to electronically share their PHI (ie, HIE) for provision of care, health system planning, REB-approved research, and patient-mediated exchange (*behavioral reaction*).

### Data Analysis

The data analysis was conducted independently by 2 authors (NS and LS). At various points throughout the process, the authors compared their analysis and resolved any disagreements through discussion. NVivo 9 (QSR International) qualitative analysis software was used to code and organize the data.

**Figure 2:**
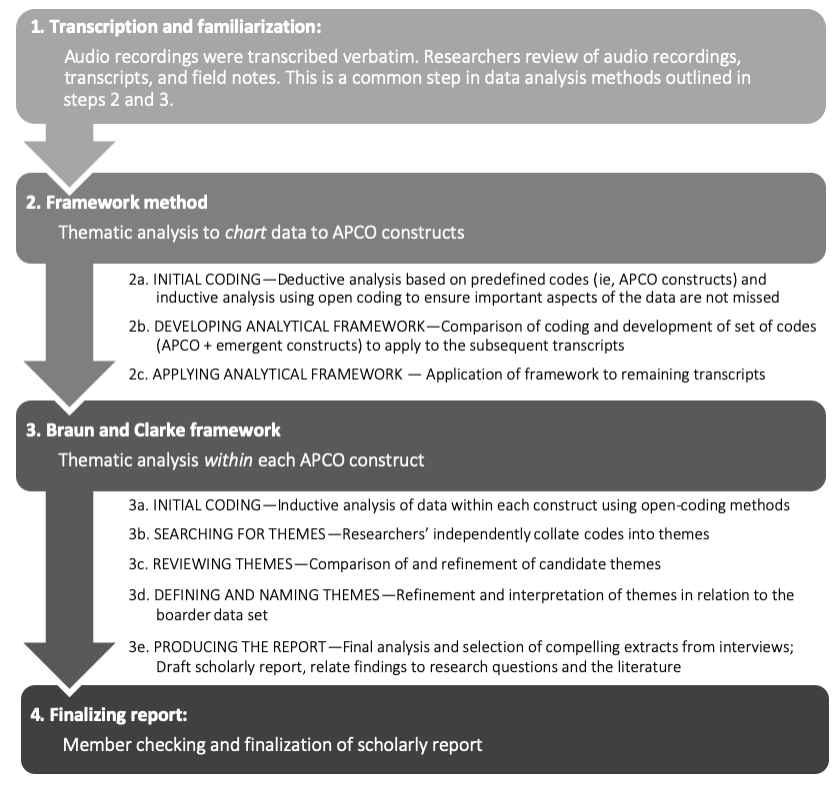
Approach to data analysis. APCO: Antecedent-Privacy Concern-Outcome.

A thematic analysis of the data was conducted in 2 phases using the framework method [62] and Braun and Clarke framework (Figure 2) [63,64]. The framework method [62] was used in the first phase to *chart* the data to the APCO. The data were deductively analyzed using the APCO constructs as predefined codes [65]. Open coding was used when data did not fit the predefined codes, and themes were inductively generated. This allowed for the extension of the APCO by uncovering health care–specific concepts (or constructs) not captured in the original model [62,66,67]. The Braun and Clarke thematic analysis framework [63,64] was used in the second phase to inductively analyze the data collated within each construct. After achieving consensus between the 2 authors, a final report was drafted where selected extracts relating to the analysis and the research questions were highlighted. This report was circulated to participants via email for member checking to ensure the accuracy and credibility of the reported results [68]. We did not receive any conflicting or discrepant feedback from the participants. As such, the findings were finalized and reported in this paper.

## Results

### Participant Characteristics

A total of 47 patients inquired about the study, of which 21 patients completed the preinterview questionnaire. On the basis of their questionnaire responses, 4 participants were excluded from the study because they did not add heterogeneity on the trusting dispositions subscales and did not report any patient characteristics unique to the sample. In total, 17 unique participants were included in the study; however, 3 participants were not interviewed as they were lost to follow-up. The characteristics of the 14 participants are reported in Table 2. Most participants were daily internet users and used the internet for health-related purposes. Participants generally had a trusting disposition. All participants trusted in the competence of others, and only 1 participant did not have a trusting stance.

**Table 2:** Participant characteristics.

Characteristics			Number of participants (n)
**CAMH^a^ program**			
	Addiction Medicine Services		6
	Mood & Anxiety Services		8
	Both		1
**Years at CAMH**			
	<1		7
	>1		7
**Gender**			
	Male		7
	Female		7
	Other options^b^		0
**Age (years)**			
	18-34		3
	35-44		5
	45-64		5
	>65		1
**Self-rated health status**			
	Poor		0
	Fair		5
	Good		8
	Very good		1
	Excellent		0
**Internet use**			
	At least once a day		13
	At least once a week		1
	At least once a month		0
	Less than once a month		0
**Trusting disposition**			
	Trust		10
	Neutral		2
	Distrust		2
**Integrity**			
	Trust		9
	Neutral		4
	Distrust		1
**Competence**			
	Trust		14
	Neutral		0
	Distrust		0
**Trusting stance**			
	Trust		13
	Neutral		0
	Distrust		1
**Type of internet use**			
	**Health-related uses**		11
		Search for health info	7
		Use of health information technology^c^	4
		Email health care provider	2
	Personal		10
	Information seeking		9
	Entertainment		8
	Tasks and services		8
	Purchasing		6

^a^CAMH: Centre for Addiction and Mental Health.

^b^Other options provided included the following: trans-sexual, transgendered, gender-queer, 2-spirit, female-to-male, male-to-female, intersex, unsure, questioning, prefer not to answer, other (please specify).

^c^Access their lab results, manage their health records or clinical appointments, or file insurance claims.

### Patient Privacy Perspective (Privacy Concern)

Privacy was defined by some as having some control over who could access their information. Others equated privacy with confidentiality (eg, need to *protect* and limit *access* to other parties). Privacy was also normative, described as how people and PHI should be treated by using terms such as *respect, trust, appreciating, understanding*, and *honesty*.

There was an agreement that privacy was important in health care—often referring to privacy as a patient right. Privacy is especially important because of the stigma associated with mental health. Without privacy, discrimination may *hamper their ability to do [things]* and *prevent them from living a fulfilled life*. A *professional patient* shared his experiences:

I've had HIV since the '90s and that was a concern... that information getting out in the early days because it was the plague and you're a social leper. Now mental health is that [way]. They're just labeled as crazy.

INT10

Participants were divided on whether they had privacy concerns with HIE. Although they were quick to discuss their perceived risks (ie, hackers, unauthorized access by employers, and insurance companies), it did not appear to be of *particular* concern to some. Most participants discussed past *privacy experience* as their reason for concern.

Past privacy incidents or negative experiences with family, friends, colleagues, and acquaintances were reasons why some participants were cautious about discussing their mental health with other people. A participant provided the following account:

I have, not real concerns... I've got reservations. Since I had one negative experience... [T]he insurance company sent my medical records, all of them [since birth], because of the consent form that I signed—not just for that particular incident with the bipolar illness—were sent to my president... [T]hat individual decided, “I don't want a nutcase for a VP” and did everything possible to make me quit, but I didn’t... [W]e had a long time in court, and I won. It was 100% undoubtedly proven in court.

INT4

Although many participants did not have past privacy incidents, a number of them brought up the frequent media reports about high-profile organizations being hacked, incidents at local hospitals, lost computers and Universal Serial Bus drives, and improper disposal of *obsolete computers*. Despite this heightened awareness, it was not a direct concern for most participants as they saw hacking as a new reality in the digital world—people with *nefarious intentions* will find a way to gain access to PHI regardless of the protective measures undertaken. This fatalistic view was described by a participant:

...it's happening all the time now... like I said before, you hope that it won't happen, but it could happen and that's just, I hate to say it, something you just have to get used to. (laughs) Convenience opens those doors for... breaches and things like that.

INT9

Privacy experiences within a health care setting appeared to have a more direct impact on privacy concern. Positive experiences with doctors and health care institutions handling information reassured participants that their privacy was taken seriously. In a few cases, their doctor’s candor and outlook on EHRs were able to quell their concerns:

[E]very two-three months [you hear] about something that's been hacked... On the other hand, I'm impressed with the way my doctors handle the information. Though Dr [C] says that... some things were faster with paper. But on the whole, you know, they can access information, they can do drug interactions, they can look up history, that you know, they can give out information among themselves. It's very good.

INT2

Negative health care experiences were reasons for concern. Some participants recalled incidents where patient data were visibly out in the open, openly discussed in waiting areas, or given to the wrong person. A participant shared her experiences as a patient and as clinician-researcher working at a different hospital:

[T]hat's mostly a systemic failure. There are other people like I described, like my former supervisors that, you know, are clearly flagrantly violating like privacy laws as well as just sort of, I don't know, the social contract.

INT14

The longitudinal content in the EHR was also a reason for concern for some participants. They discussed instances where they were treated differently or judged by health care providers because of what was in their records. A couple participants felt that past diagnosis, which they believe to be less relevant in the present, could still be used against them. A participant shared this concern:

Privacy is huge for me. I'm a pretty private person. I didn't realize... [that]as an inpatient, every single thing that you do and that you say, it gets... [charted] right into the, I guess now, the computer system... and it's all in there for, I guess forever for them to see... [T]hey bring it to the future where you've grown from that experience and they hold over your head for, you know, two, three years later. If you go to hearings or review boards, they bring the past with you. What they have written... in the system.

INT13

### Trusting Beliefs (Trust)

Trust was described as a mutual understanding earned and maintained through interpersonal relationships over time. Many spoke about trust in terms of a principal-agent relationship, where there is an element of *faith*, *reliance*, or *confidence* that a trustee will *do the right thing*, act with the trustor’s *best interests*, or deliver on some *expected outcome* that *was agreed upon*. Participants cited confidential relationships with health care providers and institutions as examples of trust:

I don't think you can legislate trust. I don't think you can write trust down in the same way you can privacy. Trust is, I think, more of an interpersonal, uh, concept.

INT14

Participants relied on common heuristics when deciding to trust individuals with PHI. First, they would share their PHI if they were *actively seeking* something or if sharing *served a purpose.* Sometimes, PHI sharing was *out of necessity* to gain access to mental health services or receive the proper care*.* A participant recalled:

I have been holding back some information... I mean, not anymore, but yes [it] just kind of reached a point where—I needed help (humph).

INT8

Later in the interview, they commented that it was *“not a benefit to say, ‘No, you can’t have access to, you know, all of the previous stuff.’ It’s kind of self-defeating.”*

Another common heuristic was credentials (ie, degrees, affiliations, and professional college memberships). Credentials meant that an individual has reached a certain level of competence or was *bound by a set of standards* or *code of ethics*. A participant’s comment best represented the role of credentials:

[T]he degree to which I believe it will be kept private and secure [depends on] the credentials of the people involved. I would trust like a doctor, like a Doctor of Medicine or a doctor of psychiatric medicine or a counselor, more than I might trust, say, a life coach. They're trying to do similar tasks to some degree, but a life coach, for example, may not have the same training, same experience, and may not be licensed in the same manner. [It’s like] listening to a doctor versus listening to someone on the internet.

INT6

Relationship-specific heuristics, such as reputation, familiarity, closeness, and history were considered when sharing PHI. Many stated the positive reputation of CAMH gave them confidence in their services. Some participants discussed how trust in health care providers was established over time and with repeated positive experiences:

[T]he head pharmacist, he's been working with me for the last like 25, 30 years and I always refer him to the pharmacist at whatever hospital I'm at, I just say, “Talk to Henry, he knows everything.”

INT1

These heuristics also apply to personal relationships. For instance, a participant (INT9) identified their mom, sister, and 2 best friends as the only people they would trust with PHI because of their history. They were confident that these individuals could *keep a secret* and *would not use it against* the individual*.* In addition, the information recipient needed to be open-minded to struggles of living with mental health conditions. Participants shared how poor attitudes or *bedside manner* might have a detrimental effect on their trust. A participant was hesitant to share with those who did not understand their chronic pain and mental health issues because of the past judgement they received from their family and others, including health care providers:

[there's a] lack of understanding of why, why aren't I doing more or, or why, why is it that I have been struggling for all of these years... so people don't associate that with, uh, chronic health issues, whether that's mental or physical and even less when you're “passing as” [a nondisabled person].

INT12

Sometimes, the decision to share PHI was *instinctual* or based on a *good vibe from the person.* Participants also attributed their trust and privacy views to their personalities (eg, *a private person*, an *open book*, or *not having that magical trust*). Trust also reflected the participant’s views on humanity. Generally, participants believed that people are well intentioned and are trying their best. For this reason, participants did not conflate past mishaps or non-PHI–related mistakes with trust in their health care providers. There was a belief that breaches occur because a small segment of the population is *malicious* or negligent with patient privacy. A few participants provided commentary on why profit-driven entities (eg, pharmaceutical industry) cannot be trusted. When asked what corporations and controversial entities can do to rebuild trust, most believed that these entities need to become transparent about their motives for how PHI would be used and what is being done to ensure its security.

### Privacy Policy and Legislation (Regulation)

Laws were seen as a form of accountability for those who handled their PHI, serving as a deterrent for improper access or unauthorized disclosure. Without laws, participants would only seek care in urgent and emergency situations. Despite the importance of law, participants had a *vague understanding* of the legislated patient privacy rights and PHI uses:

This is sort of tied into why I’m interested in this. Because one of my emergency visits a few years ago, they ended up suspending my driver's license for health issues (laughs). And it all kind of happened without me knowing, until I get a letter in the mail from the [Ministry of Transportation] saying “Your license has been suspended.” They didn’t even tell me in the hospital... so it’s kinda tied into stuff like that... Should police get access to it?... Yes or no, and then when and why?

INT3

Much of their knowledge of privacy laws and policies came from instances where they exercised certain rights. Some rights, such as the right to access their PHI, to request an audit of who accessed their PHI, and ability to place blocks on certain parts of their PHI, were interesting to participants as they felt it would have helped them in the past and could be useful in the future. Most participants *suspected* or *assumed* that PHI was used by the government for health systems planning and REB-approved research but were unfamiliar of the protective measures taken for these data (ie, prescribed entities, deidentification, and aggregation).

Participants felt reassured that much thought went into law development; however, it did not change how they felt about their PHI privacy. Some reflected on their experiences dealing with bureaucracy when exercising their rights, as a participant (INT4) noted, “that is the law, but it doesn't work that way.” Others reiterated the fatalistic belief, bringing up examples of PHI snooping of local public figures by privacy-trained health care professionals. To them, laws can only do so much as there will always be a *snoopy sally.*

Participants generally felt the government and health care institutions were responsible in protecting their privacy by establishing the privacy laws (or policies), oversight, and enforcement of those laws. Many also felt that anyone handling PHI should be responsible. A few participants accepted responsibility for themselves, explaining they should be cautious when disclosing information; however, the responsibility shifts to the health care provider once the information is disclosed. A participant quipped:

Well once you give them [your PHI], I don't know if there's a lot the patient can really do. Um, supposed to stay 'til the office closes to make sure they lo-, shut down the computer, or that its password protected?... or to make sure if they still use old paper files. Is the, is the file room locked at night? (laughs) Is there, is there a good lock on the door? Or no windows, and do they have bars on the windows?... So yeah, I think it's mostly up to the organization.

INT3

When asked what could be done to ease any concerns about the electronic sharing of PHI, many felt there was a need for more effective communication of privacy laws, recommending patient-accessible documents, such as a *top-ten list* or a *bill of rights.* Suggestions on content include simple communication (eg, “your privacy is ensured” and announcements of privacy certifications and accreditation), lists of PHI uses and protections, and a guide on how to exercise privacy rights. Some participants also suggested more active dissemination approaches, such as greater prominence on institutional websites, news features (eg, television, Web, and newspaper), and town hall meetings.

### Health Information Exchange Attitudes (Behavioral Reactions)

Participants were willing to allow their PHI to be used for clinical use, patient-mediated exchange, health service planning, and REB-approved research. A few participants voiced preferences on who could access their records and whether consent should be required; however, they were still supportive as they saw utility in the exchanges. Participants were aware of the wide range of potential benefits of PHI use. They quickly rationalized how each case could be beneficial. The *privacy calculus* was discussed in a few interviews, where *the advantages outweigh the disadvantages*; however, participants discussed the benefits and seldom discussed the risks.

Sharing PHI for clinical use was seen as advantageous, as complete information was required for the *best care possible*. Many discussed the importance of complete medical history in emergency situations and mental health crises where they were *not in a 100% sound state* or lacked capacity to discuss their medical histories. Even in nonemergency situations, clinician access to complete records can take the *stress off the patient* to remember everything or have to repeat *the same story*. They believed there would always be *gaps* in their memories regardless of how organized they are with their records.

Overall, patient-mediated exchange was thought to be a good idea but not necessarily for everyone. Some were amused by the idea and were curious to try it out. Others indicated they already used patient portals or were invited to register for access. Having access was seen by many as a way for them to review and keep track of their records, help them better understand their health, or become *partners in their care*. A few felt that having access to their PHI was a form of patient accountability, as it would allow them to refer to documentation about decisions made, ensure their PHI is accurately recorded and mistake-free, and identify which health care professionals have accessed their PHI (if possible).

Participants supported HIE for health system planning and REB-approved research, where PHI was deidentified or aggregated. There was a sense of altruism when it came to using PHI for health system planning, as it was a way for participants to *give back*, contribute to a *greater good, or* help fix a *fractured system.* They explained the government needed *reliable numbers* to address health care issues (ie, underfunded and understaffed programs, wait times, budget constraints, and access to mental health care) and plan for a more efficient or effective system. Similarly, REB-approved research was seen as beneficial and essential in finding new or better treatments, medications, and cures. The *professional patient* reflected on his medical history:

I was part of the early days of HIV. And [those] days are guinea pigs for drugs. So perhaps if, um, more information we share, more things would have come out...now that there's electronic data that's able to be shared, things are shared quicker. Who knows what advances in research would happen.

INT10

## Discussion

### Principal Findings

We conducted this study to begin bridging the patient privacy evidence gap in mental health HIE [38]. This study sought to understand the privacy perspectives (*privacy concern*) of patients with mental health conditions and explore the interplay of their perspectives with the antecedents and outcomes delineated in the APCO (ie, *trust, regulation, and behavioral reaction*). Through inductive and deductive analysis, this study introduces evidence on the context-dependency on the patient privacy perspective on mental health HIE. Although all participants agreed on the fundamental importance of privacy in health care, especially in mental health care, the degree of concern expressed in the interviews varied. Privacy concerns commonly stemmed from negative health care privacy experiences and negative health care perceptions based on their patient experience, whereas other privacy antecedents were infrequently discussed.

*Privacy experience* is a construct seldom explored in HIE patient privacy research [38]. In our findings, *privacy experience* was the only antecedent that consistently identified in the data. Although many participants were concerned about increasing occurrences of privacy breaches as reported in the press, it was not a direct concern because of their fatalistic privacy view—a belief that that breaches are a reality in our digital society, and all they could do is trust those involved will do their best to protect patient privacy. Conversely, direct experiences or observations of lack of privacy vigilance within a health care setting left a lasting impression on participants. Poor patient experiences unrelated to privacy also had the effect of leaving participants with a negative perception of the health care environment. As such, health care perceptions should be included as a construct in future adaptations of the APCO, as it was a cross-cutting theme across *privacy concern, trust, regulation,* and *behavioral reaction*.

Participants used credentials and relationship-specific heuristics to determine their comfort in sharing about their mental health with others (*trust*). They generally trusted that health care professionals and institutions would protect the privacy of any information shared in receiving care. This degree of trust is accentuated by a lack of knowledge about the legislated PHI uses (*regulation*), especially when juxtaposed with the high importance they placed on law. There was a passive acceptance that legislative and institutional safeguards would ensure those working with PHI are properly trained and accountable to their conduct. Whether privacy related or not, poor patient experiences (eg, bureaucracy and bedside manner) caused skepticism about the effectiveness of the legislative and institutional safeguards protecting their privacy. This is consistent with other studies, where patient perceptions of quality of care, patient-physician relationship, and trust in health care providers have strong associations with perceptions of privacy and PHI sharing attitudes [38,69-71].

Despite the varying perspectives on privacy and trust, participants were pragmatic about HIE and its potential PHI uses (ie, *behavioral reaction*), recognizing the best care required the best information possible. Some participants reflected on their experiences in accessing and receiving mental health care or perceptions about the health care system, acknowledging that sharing PHI is necessary to improve treatments and health care policy decisions through research and analytics. The patient-mediated exchange was novel to some participants; however, they understood the value of accessing and managing their records and agreed that interested patients should have the option to do so. These individual and societal benefits of HIE were the primary focus in most responses to *behavioral reaction* questions, whereas the risks of HIE were seldom discussed. As suggested earlier, participant-*perceived risks* might have been muted by their fatalistic privacy views. Receiving the best care possible may also supersede the need for their personal risk assessment [72].

Echoing past policy recommendations [39,73-77], participants suggested the following as the first steps in fostering trust: transparent communication of the value of interoperable HIT, PHI uses, protective measures, and patient privacy rights. In addition to public education, patient engagement is essential to its success [78-80]. Patient feedback is critical in the highly debated topic of consent [81-84]. Surprisingly, consent was rarely mentioned by participants, especially as studies found patients wanted granular control of their PHI [85,86]. Their passive acceptance and pragmatic views suggest that contextual integrity may be a viable alternative approach to the consent. Contextual integrity assumes the act of sharing information is only an issue when shared outside the boundaries of socially acceptable contextual norms (ie, *norms of appropriateness* and *norms of flow*) [87,88]. These contextual norms provide a technology-agnostic standard to evaluate the acceptability of new HIT, as they capture the patients’ perspectives with respect to information flow. Patient engagement and deliberation on PHI privacy will be required to establish these norms [89].

Finally, understanding the patient health care perceptions can provide privacy and HIT policy and decision makers with insights on where health care system exceeds or fails to meet their privacy expectations. These insights inform how the health care environment, processes, and delivery can be redesigned to foster greater patient trust and mitigate their concerns. Addressing privacy concerns in a way that is vigilant and sensitive to the health care environment can improve patient views on privacy and patient satisfaction [90]. These improvements will require a strong commitment to making major administrative, philosophical, and operational changes that respect both patient privacy and satisfaction.

### Limitations

This study contributes to the understanding of the privacy perspectives of patients with mental health conditions or sensitive PHI—an area where there is a dearth of research. Limitations on the sample and study context should be considered when interpreting the findings. First, the challenges of recruiting patients in a mental health setting may have limited the sample size; however, studies have shown that data saturation can be achieved with sample sizes anywhere from 12 to 17 participants [91,92]. Our findings may not reflect the views of the broader mental health population, including patients receiving care in other CAMH programs and services. Although we employed a maximum variation sampling strategy, the findings are not intended to be generalizable nor numerically representative; rather, this sampling strategy is intended to highlight diversity in responses [93].

The results of this study may reflect the views of patients with more trusting dispositions, as there was difficulty identifying participants with distrusting stances. Given distrust is a predisposing factor of health care avoidance [94], those receiving care may be more trusting or become more trusting because of their positive experiences at CAMH. As CAMH is an academic hospital, participants may be more familiar with the health care system and how their PHI is used for health system analytics and research. Moreover, these findings pertain to the Canadian context and may not be applicable to other countries. In Canada, the *universal health care* system is a part of the national identity [20], which may influence participant awareness or understanding of its sustainability. Canadians may have a more favorable health care perceptions and views of HIE, which could positively bias their *behavioral reaction*.

The use of a proxy measure for achieving maximum variation in trusting disposition should be considered. As observed in the interviews, the degree in which participants trusted others with their PHI varied from trusting no one to trusting everyone bound by privacy law. These differences in responses in the interviews indicated variation was achieved with trust. These differences also suggest the trusting disposition scale may not have been appropriate for this study, as it was rigorously validated in a nonhealth care context (ie, electronic commerce) [58]. The role of trust in patient participation in research may be another explanation for the difficulty in recruiting distrusting participants. Using trust as a parameter for variation may introduce self-selection bias, as trusting patients may be more willing to participate in research [95-97]. As the trusting disposition scale was related to participants’ *personality*, the observer bias (ie, Hawthorne effect) should also be considered [98]. Participants in active care may not be fully candid with their views on how their PHI is being handled, given the research team’s affiliation with CAMH. Efforts were made at every step of this study to ensure that the patients understood that the study is independent to the care they received and their individual responses would remain anonymous.

Finally, the privacy perspectives in this study includes those who work in health care or research. Although their views may include professional insights on PHI privacy, being a patient does not preclude privacy experiences from other facets of life as delineated in the APCO. The findings reported here represent views echoed by other participants and were identified through thematic analysis.

### Conclusions

Through their first-hand accounts, this study introduces evidence that patients with mental health conditions support HIE in Canada, where the benefits to their health was compelling enough to overcome privacy concerns over the risks associated with sharing their PHI. Patients saw the societal value of sharing their potentially stigmatizing PHI to support the single-payer universal Canadian health care system. Their fatalistic view on digital information underscores the importance of trust in the patient privacy discussion. Although these findings are within the Canadian context, this study highlights how engaging patients can illuminate the nuances to the patient privacy perspective that are often lost in mental health privacy conjecture. The nuances associated with trust and the patient experience are seldom explored in the HIE privacy discourse; however, these are critical in reassuring patients that the health care system prioritizes patient privacy in providing the best care possible. With many innovative and transformative PHI uses on the horizon, it is imperative that health care systems globally engage patients to ensure that patient-centric privacy policy decisions about PHI are made and are reflective of the nuanced views of the patients.

## Appendix

Multimedia Appendix 1 Pre-interview questionnaire.

Multimedia Appendix 2 Interview Guide.

## Abbreviations

APCO: Antecedent-Privacy Concern-Outcome
CAMH: Centre for Addiction and Mental Health
EHR: electronic health record
HIE: health information exchange
HIT: health information technology
PHI: personal health information
REB: research ethics board
