# Supplementary material for: Patient Privacy Perspectives on Health Information Exchange in a Mental Health Context: Qualitative Study
Source: JMIR Ment Health. 2019 Nov 13;6(11):e13306. doi: 10.2196/13306 (PMC6881785; doi:10.2196/13306)
Supplement: Multimedia Appendix 1 [file mental_v6i11e13306_app1.pdf]

## Pre-Interview Questionnaire

1. At which CAMH program are you currently receiving care?
   a. Addictions Medicine Services
   b. Ambulatory Addictions Services
   c. Mood and Anxiety
   d. Other:___________________________

2. What is your gender?
   a. Male
   b. Female
   c. Transsexual
   d. Transgendered
   e. Gender-queer
   f. Two-spirit
   g. Female to Male
   h. Male to Female
   i. Intersex
   j. Unsure
   k. Questioning
   l. Prefer not to answer
   m. Other:_______________________________________

3. What is your Age?
   a. 18-34
   b. 35-44
   c. 45-64
   d. >65

4. How do you rate your health status (including mental health) in general?
   a. Poor
   b. Fair
   c. Good
   d. Very Good
   e. Excellent

5. How often do you use the internet for personal use in a typical month?
   a. At least once a day
   b. At least once a week (but not every day)
   c. At least once a month (but not every month)
   d. Less than once a month

6. What do you use the internet for (circle all that apply)?
   a. Purchasing (e.g., online shopping)
   b. Information Seeking (e.g., news, hobbies, health, etc.)
   c. Entertainment (e.g., music, movies, games)
   d. Task/Services (e.g., banking, bills, personal information tasks, etc.)
   e. Personal (e.g. Emails, instant messaging, social media)

7. Do you use the internet for any health-related activities?
    a. Yes
    b. No
    c. If yes, please specify: ________________________

8. In general, people really do care about the well-being of others
    a. Strongly Disagree
    b. Disagree
    c. Neither Disagree or Disagree
    d. Agree
    e. Strongly Agree

9. The typical person is sincerely concerned about the problems of others
    a. Strongly Disagree
    b. Disagree
    c. Neither Disagree or Disagree
    d. Agree
    e. Strongly Agree

10. Most of the time, people care enough to try to be helpful, rather than just looking out for themselves.
    a. Strongly Disagree
    b. Disagree
    c. Neither Disagree or Disagree
    d. Agree
    e. Strongly Agree

11. In general, most folks keep their promises
    a. Strongly Disagree
    b. Disagree
    c. Neither Disagree or Disagree
    d. Agree
    e. Strongly Agree

12. I think people generally try to back up their words with their actions
    a. Strongly Disagree
    b. Disagree
    c. Neither Disagree or Disagree
    d. Agree
    e. Strongly Agree

13. Most people are honest in their dealings with others
    a. Strongly Disagree
    b. Disagree
    c. Neither Disagree or Disagree
    d. Agree
    e. Strongly Agree

14. I believe that most professional people do a very good job at their work
    a. Strongly Disagree
    b. Disagree
    c. Neither Disagree or Disagree
    d. Agree
    e. Strongly Agree

15. Most professional are very knowledgeable in their chosen field
    a. Strongly Disagree
    b. Disagree
    c. Neither Disagree or Disagree
    d. Agree
    e. Strongly Agree

16. A large majority of professional people are competent in their area of expertise
    a. Strongly Disagree
    b. Disagree
    c. Neither Disagree or Disagree
    d. Agree
    e. Strongly Agree

17. I usually trust people until they give me a reason not to trust them
    a. Strongly Disagree
    b. Disagree
    c. Neither Disagree or Disagree
    d. Agree
    e. Strongly Agree

18. I generally give people the benefit of the doubt when I first meet them
    a. Strongly Disagree
    b. Disagree
    c. Neither Disagree or Disagree
    d. Agree
    e. Strongly Agree

19. My typical approach is to trust new acquaintances until they prove that I should not trust them.
    a. Strongly Disagree
    b. Disagree
    c. Neither Disagree or Disagree
    d. Agree
    e. Strongly Agree
