# Supplementary material for: Patient Privacy Perspectives on Health Information Exchange in a Mental Health Context: Qualitative Study
Source: JMIR Ment Health. 2019 Nov 13;6(11):e13306. doi: 10.2196/13306 (PMC6881785; doi:10.2196/13306)
Supplement: Multimedia Appendix 2 [file mental_v6i11e13306_app2.pdf]

Client/Patient Interview Script

BACKGROUND SCRIPT:
The use of electronic personal health information is an important issue for patients, healthcare workers, and researchers. While there are Federal, Provincial, and institutional laws and policies to protect the privacy and confidentiality of patients, we are becoming increasingly sensitive to the importance of consulting with patients about their views, especially with many new innovations on the horizon that may improve the quality of our healthcare system.
The purpose of my study is to understand your views on how your personal health information is and should be used. Some questions may be stressful for you to answer; however, feel free to skip the question by saying 'pass' if you do not want to answer. You may also end the interview at anytime if you feel any discomfort with the questions. Please do not hesitate to ask questions if you have any.

INTERVIEW QUESTIONS:

Privacy and Trust
(SCRIPT: We'll start off with a few questions asking for your thoughts on what privacy and trust mean to you in the context of your personal health information and the health care system)

1. What does privacy mean to you?/ How would you define privacy?

2. Is privacy important in healthcare? Why?

3. Do you have any concerns about the privacy of your personal health information?
    - If so, what are you concerned about?
    - If no, why are you not concerned?

4. What does trust mean to you? / How would you define trust?

5. What factors play a role in your decision to trust an individual with your personal health information?

Regulations
(*SCRIPT*: This set of questions will ask you for your thoughts about how your personal health information is protected by the law and institutional policies)

1. If there were no laws, would you still trust the healthcare system with your personal health information?
    - Why or Why not?

2. Who do you feel is responsible for protecting your electronic personal health information?

3. What is your current understanding of how your health information is protected?
   - What aspects are you aware of?
   - Does it affect the types of information you're willing to share? How?

   Share high-level concepts within the privacy taxonomy (under development) that was not brought up in the previous questions.
   - i. *Right to access and own a copy of own records*
   - ii. *Right to know who has accessed your records*
   - iii. *Only used for provision of care – can't share with others ulsess there is consent*
   - iv. *Consent Directives*
   - v. *Health System Planning*
   - vi. *REB-Research*

4. How does knowing these provisions make you feel more protected?

5. What can be done to ease concerns about the electronic sharing of your personal health information?

TRUSTING BELIEFS
(*SCRIPT*: The next set of questions will focus on whom you would trust with your personal health information. This could be people or institutions.)

1. Who do you trust with access to your personal health information (i.e., who do you think will keep it confidential)?
   - Why?

2. Who don't you trust with access to your personal health information (i.e., who do you think will disclose information without your authorization)?
   - Why?
   - What can these individuals (or institutions) do to build trust?

(Script: This final set of questions will ask you for your thoughts on how your health information should be used)

3. Would you share your medical information electronically between doctors, healthcare providers, hospitals, and other places you receive care? Why or why not?

4. Would you share your health information electronically for medical/healthcare research? Why or why not?

5. Would you like the ability to electronically access your medical information online? Why or why not?

PROBE (for Q1-3): Ask about awareness of the potential benefits of the three mechanism of HIE and whether that changes their willingness to share/access

6. What impact does controversies (such as the eHealth Ontario scandal) have on your trust in the healthcare system managing your personal health information?
    a. If neutral or positive, ask why they feel that way.
    b. If negative, what can be done to re-establish your trust in the system?
